# Malaria Control in South America— Response to P.C. Matteson

**DOI:** 10.3201/eid0502.990230

**Published:** 1999

**Authors:** Donald R. Roberts, Larry L. Laughlin

**Affiliations:** The Uniformed Services University of the Health Sciences, Bethesda, Maryland, USA


**Patricia C. Matteson**
U.N. Food and Agriculture Organization Programme for Community Integrated Pest Management in Asia, Hanoi, Vietnam

### References

1. Robert DR, Laughlin LL, Hshieh P, Legters LJ. DDT, global strategies, and a malaria control crisis in South America. Emerg Infect Dis 1997:3:295-302.
2. Wirth DF, Cattani J. Winning the war against malaria. Technology Review 1997;Aug/Sep:52-61.
3. World Resources Institute, United Nations Environment Programme, United Nations Development Programme, the World Bank. World Resources 1998-99. New York: Oxford University Press; 1998.
4. Matteson PC, editor. Disease vector management for public health and conservation. Washington: World Wildlife Fund-US; 1999.
5. Wania F, Mackay D. Tracking the distribution of persistent organic pollutants. Environmental Science & Technology News 1996;30:390-6.
6. Bouwman H, Cooppan RM, Becker PJ, Ngxongo S. Malaria control and levels of DDT in serum of two populations in Kwazulu. Journal of Toxicology and Environmental Health 1991;33:141-55.
7. Rogan WJ, Gladen BC, McKinney JD, Carreras N, Hardy P, Thullen J, et al. Polychlorinated biphenyls (PCBs) and dichlorodiphenyl dichloroethan (DDE) in human milk: effects on growth, morbidity, and duration of lactation. Am J Public Health 1987;177:1294-7.
8. Gladen BC, Rogan WJ. DDE and shortened duration of lactation in a northern Mexican town. Am J Public Health 1995;85:504-8.
9. Toxicological profile for 4,4'-DDE, 4,4'-DDD (updated). Atlanta: U.S. Department of Health and Human Services, Public Health Service, Agency for Toxic Substances and Diseases Registry; 1994. Pub. no. TP-93/05.
10. Eriksson P. Developmental neurotoxicity of environmental agents in the neonate. Neurotoxicology 1997;48:5719-26.
11. Rehana T, Rao PR. Effect of DDT on the immune system in Swiss albino mice during adult and perinatal exposure: humoral responses. Bull Environ Contam Toxicol 1992;48:535-40.
12. Banerjee BD, Saha S, Mohapatra TK, Ray A. Influence of dietary protein on DDT-induced immune responsiveness in rats. Indian J Exp Biol 1995;33:739-44.
13. Ray DE. Pesticides derived from plant and other organisms. In: Hayes WJ, Laws ER, editors. Handbook of pesticide toxicology. Vol 2. San Diego (CA): Academic Press; 1991. p. 585-636.
14. Casida JE, Gammon DW, Glickman AH, Lawrence LJ. Mechanisms of selective action of pyrethroid insecticides. Annu Rev Pharmacol Toxicol 1983;23:413-38.
15. Patro N, Misha SK, Chattopadhyay M, Patro IK. Neurological effects on deltamethrin on the postnatal development of cerebellum of rat. Journal of Biosciences 1997;22:117-30.
16. Smolen MJ, Sang S, Liroff RA. Hazards and exposures associated with DDT and synthetic pyrethroids used for vector control. Washington: World Wildlife Fund-US;1999.
17. Resolving the DDT dilemma: protecting biodiversity and human health. Toronto, Canada: World Wildlife Fund-Canada; 1998.

### Malaria Control in South America—Response to P.C. Matteson

**To the Editor:** Dr. Matteson, whose letter relies heavily on unpublished information and nonrefereed publications, states that growing drug resistance has contributed to increasing malaria. While drug resistance is important, when DDT use declined below effective levels (1), the proportion of *Plasmodium falciparum* infections (including infections with resistant strains) compared with *P. vivax* infections (no resistance) did not progressively increase (2). Moreover, malaria has increased in Central America, where drug resistance is unknown (3-6). As for attributing increasing malaria to deteriorating public health systems, the changes imposed on developing countries (in organizational structures of malaria control programs and prohibiting DDT [1,7]) correlate with increasing malaria rates (1).

Dr. Matteson states that large-scale migration explains why almost all Brazilian malaria cases occur in the Amazon Basin. However, DDT cleared malaria from the more populated and temperate southern regions of the country (8, unpublished report: U.S. Agency for International Development review in 1973-74 of Brazil's malaria eradication program). When DDT was in full use (pre-1980), large increases in malaria did not accompany population movement (1). With the 1970s' colonization program of the Basin came malaria problems, but not large population-based malaria increases. DDT prevented that (1,9-11). However, since DDT has been eliminated, persistent urban malaria is again becoming a problem (12-16).

Other factors (biting behavior, housing conditions, and human behavior), which Dr. Matteson attributes to increasing malaria, have always thwarted interdiction of malaria transmission in the Amazon Basin (17;18; an unpublished report: U.S. Agency for International Development review in 1973-74 of the malaria eradication program in Brazil) and are no more important today than they were before.

A UN-facilitated global negotiation process cited as a meaningful debate for malaria control is an effort to provide a legally binding agreement for global elimination of DDT and other persistent organic pollutants, not an open forum for debate of DDT use for malaria control.

Dr. Matteson claims that DDT is associated with reduced lactation. In the United States, where DDT has been banned for 26 years, mothers who stay home breast-feed for an average of 25.1 weeks—mothers who work parttime, for 22.5 weeks (19). In Belize, mothers in urban areas, where DDT is not used for malaria control, breast-feed less than 38.4 weeks—mothers in rural areas with lifetime exposures to DDT breast-feed more than 57.2 weeks (20).

The World Wildlife Fund's mass balance model of DDT sprayed in houses used to refute our assessment that DDT does not readily move away from sprayed houses also mentions that "There are few...data against which to validate the results of this...model, although actual data...should not be difficult to obtain." (21). Studies of DDT use in agriculture show that most DDT settles where it is applied (22).

Studies have shown no meaningful population-based adverse health effects from DDT use, despite more than 50 years' exposure, and evidence argues forcefully that DDT does not cause breast cancer (23).

**Donald R. Roberts and Larry L. Laughlin**
The Uniformed Services University of the Health Sciences, Bethesda, Maryland, USA

### References

1. Roberts DR, Laughlin LL, Hsheih P, Legters LJ. DDT, global strategies, and a malaria control crisis in South America. Emerg Infect Dis 1997;3:295-302.
2. Brasil. Registro de casos de malária—1960 a 1997. Gerência Técnica de Malária/FNS-Brasilia, Brasilia, Brasil.
3. Pan American Health Organization. Status of malaria programs in the Americas. XL report. Washington: The Organization; 1991. p. 145.
4. Pan American Health Organization. Status of malaria programs in the Americas. XLII report. Washington: The Organization; 1994. p. 116.
5. Pan American Health Organization. Status of malaria programs in the Americas. XLIII report. Washington: The Organization; 1995. p. 25.
6. Pan American Health Organization. Status of malaria programs in the Americas. XLIV report. Washington: The Organization; 1996. p. 23.
7. Roberts DR. Resurgent malaria: DDT and global control. U.S. Medicine 1998;34:36-8.
8. de Bustamante FM. Distribuição geográfica e periodicidade estacional da malária no Brasil e sua relação com os fatores climáticos. Situação atual do problema. Revista Brasileira de Malariologia e Doenças Tropicais 1957;9:181-90.
9. Pinheiro FP, Bensabath G, Rosa APAT, Lainson R, Shaw JJ, Ward R, et al. Public health hazards among workers along the Trans-Amazon Highway. Journal of Occupational Medicine 1977;19:490-6.
10. Smith NJH. Colonization lessons from a tropical forest. Science 1982;13:755.
11. Roberts DR. Health problems of colonists. Science 1982;217:484.
12. Sandoval JJF, Diniz R, Saraiva MGG, da Silva EB, Alecrim WD, Alecrim MGC, et al. Histórico da malária na cidade de Manaus e proposta de controle integrado. Rev Soc Bras Med Trop 1998;31, Suplemento 1:141.
13. Amaral JCOF, Machado RLD, Segura MNO, Oliveira GS, Povoa MM. Avaliação longitudinal da infecção causada por *Plasmodium falciparum* e/ou *Plasmodium vivax* na população de duas localidades de Icoaraci, Distrito de Belem, Para. Rev Soc Bras Med Trop 1998;31 Suplemento 1:16.
14. da Silva EB, Costa MF, Melo YFC, Alecrim MGC. Inquérito soroepidemiologico numa área urbana em fase de ocupação, na cidade de Novo Aryao-Amazonas-Brasil. Rev Soc Bras Med Trop 1998;31 Suplemento 1:82.
15. Ventura AM, Pinto AY, Uchoa R, Calvosa V, Santos MA, Filho MS, et al. Malária por *Plasmodium vivax* em crianças-I-aspectos epidemiologicos e clínicos. Rev Soc Bras Med Trop 1998;31 Suplemento 1:82.
16. Suarez MC, Fe NF, Alecrim WD. Estudo do processo de transmissão da malária em uma área de invasão recente na cidade de Manaus Amazonas. Estudo entomologico. Rev Soc Bras Med Trop 1998;31 Suplemento 1:15-6.
17. Forattini OP. Entomologia medica: I volume parte Geral, Diptera, Anophelini. São Paulo (Brasil): Faculdade de Higiene e Saúde Publica; 1962. p. 414.
18. Rachou RG. Some manifestations on behaviouristic resistance in Brazil. Semina Suscep. Insects to insecticides, Panama, Report.: WHO 1958:208-95.
19. Frank E. Breastfeeding and maternal employment: two rights don't make a wrong. Lancet 1998;352:1083-4.
20. Central Statistical Office, Belize. 1991 Belize family health survey, final report. Reprinted by U.S. Dept of Health and Human Services; 1992. p. 69.
21. Resolving the DDT dilemma: protecting biodiversity and human health. Toronto, Canada: World Wildlife Fund-Canada; 1998.
22. World Health Organization. DDT and its derivatives. Environmental health criteria 9. Geneva: The Organization; 1979. p. 194.
23. Safe, SH. Xenoestrogens and breast cancer. N Engl J Med 1997;337:1303-4.

### On the Etiology of Tropical Epidemic Neuropathies

**To the Editor:** In a recent report of an epidemic of optic neuropathy in Dar es Salaam, Tanzania (1), Dolin et al. state that the disease is clinically identical to one of the forms of epidemic neuropathy found in Cuba between 1991 and

**To the Editor:** Dr. Matteson, whose letter relies heavily on unpublished information and nonrefereed publications, states that growing drug resistance has contributed to increasing malaria. While drug resistance is important, when DDT use declined below effective levels (1), the proportion of *Plasmodium falciparum* infections (including infections with resistant strains) compared with *P. vivax* infections (no resistance) did not progressively increase (2). Moreover, malaria has increased in Central America, where drug resistance is unknown (3-6). As for attributing increasing malaria to deteriorating public health systems, the changes imposed on developing countries (in organizational structures of malaria control programs and prohibiting DDT [1,7]) correlate with increasing malaria rates (1).

Dr. Matteson states that large-scale migration explains why almost all Brazilian malaria cases occur in the Amazon Basin. However, DDT cleared malaria from the more populated and temperate southern regions of the country (8, unpublished report: U.S. Agency for International Development review in 1973-74 of Brazil's malaria eradication program). When DDT was in full use (pre-1980), large increases in malaria did not accompany population movement (1). With the 1970s' colonization program of the Basin came malaria problems, but not large population-based malaria increases. DDT prevented that (1,9-11). However, since DDT has been eliminated, persistent urban malaria is again becoming a problem (12-16).

Other factors (biting behavior, housing conditions, and human behavior), which Dr. Matteson attributes to increasing malaria, have always thwarted interdiction of malaria transmission in the Amazon Basin (17;18; an unpublished report: U.S. Agency for International Development review in 1973-74 of the malaria eradication program in Brazil) and are no more important today than they were before.

A UN-facilitated global negotiation process cited as a meaningful debate for malaria control is an effort to provide a legally binding agreement for global elimination of DDT and other persistent organic pollutants, not an open forum for debate of DDT use for malaria control.

Dr. Matteson claims that DDT is associated with reduced lactation. In the United States, where DDT has been banned for 26 years, mothers who stay home breast-feed for an average of 25.1 weeks—mothers who work parttime, for 22.5 weeks (19). In Belize, mothers in urban areas, where DDT is not used for malaria control, breast-feed less than 38.4 weeks—mothers in rural areas with lifetime exposures to DDT breast-feed more than 57.2 weeks (20).

The World Wildlife Fund's mass balance model of DDT sprayed in houses used to refute our assessment that DDT does not readily move away from sprayed houses also mentions that "There are few...data against which to validate the results of this...model, although actual data...should not be difficult to obtain." (21). Studies of DDT use in agriculture show that most DDT settles where it is applied (22).

Studies have shown no meaningful population-based adverse health effects from DDT use, despite more than 50 years' exposure, and evidence argues forcefully that DDT does not cause breast cancer (23).
